# Trajectories of objectively measured physical activity and childhood overweight: longitudinal analysis of the IDEFICS/I.Family cohort

**DOI:** 10.1186/s12966-021-01171-2

**Published:** 2021-08-09

**Authors:** Ole Sprengeler, Hermann Pohlabeln, Karin Bammann, Christoph Buck, Fabio Lauria, Vera Verbestel, Gabriele Eiben, Kenn Konstabel, Dénes Molnár, Luis A. Moreno, Yannis Pitsiladis, Angie Page, Lucia Reisch, Michael Tornaritis, Wolfgang Ahrens

**Affiliations:** 1 Department of Epidemiological Methods and Etiological Research, Leibniz Institute for Prevention Research and Epidemiology – BIPS, Achterstr. 30, D-28359 Bremen, Germany; 2 Department of Biometry and Data Management, Leibniz Institute for Prevention Research and Epidemiology – BIPS, Achterstraße 30, D-28359 Bremen, Germany; 3 Working group Epidemiology of Demographic Change, Institute for Public Health and Nursing Sciences (IPP), University of Bremen, Bremen, Germany; 4 Institute of Food Sciences, National Research Council of Italy, Avellino, Italy; 5 Department of Rehabilitation Sciences, Faculty of Medicine and Health Sciences, Ghent University, Ghent, Belgium; 6 Department of Public Health, School of Health Sciences, University of Skövde, Skövde, Sweden; 7 Department of Chronic Diseases, National Institute for Health Development, Tallinn, Estonia; 8 School of Natural Sciences and Health, Tallinn University, Tallinn, Estonia; 9 Institute of Psychology, University of Tartu, Tartu, Estonia; 10 Department of Paediatrics, University of Pécs, Pécs, Hungary; 11 GENUD (Growth, Exercise, Nutrition and Development) Research Group, Faculty of Health Sciences, University of Zaragoza, Edificio del SAI, C/Pedro Cerbuna s/n, 50009 Zaragoza, Spain; 12 Instituto Agroalimentario de Aragón (IA2), Zaragoza, Spain; 13 Instituto de Investigación Sanitaria Aragón (IIS Aragón), Zaragoza, Spain; 14 Centro de Investigación Biomédica en Red de Fisiopatología de la Obesidad y Nutrición (CIBERObn), Madrid, Spain; 15 Collaborating Centre of Sports Medicine, University of Brighton, Welkin House, Eastbourne, UK; 16 Centre for Exercise, Nutrition and Health Sciences, School of Policy Studies, University of Bristol, Bristol, BS8 1TZ UK; 17 NIHR Bristol Biomedical Research Centre, University Hospitals Bristol NHS Foundation Trust and University of Bristol, Bristol, UK; 18 Copenhagen Business School, Copenhagen, Denmark; 19 Research and Education Institute of Child Health, Strovolos, Cyprus; 20 Institute of Statistics, Faculty of Mathematics and Computer Science, University Bremen, Bremen, Germany

**Keywords:** Health-enhancing physical activity, Pediatric overweight, Obesity prevention, Dose-response relationship, Physical activity recommendations

## Abstract

**Background:**

Since only few longitudinal studies with appropriate study designs investigated the relationship between objectively measured physical activity (PA) and overweight, the degree PA can prevent excess weight gain in children, remains unclear. Moreover, evidence is limited on how childhood overweight determines PA during childhood. Therefore, we analyzed longitudinal trajectories of objectively measured PA and their bi-directional association with weight trajectories of children at 2- and 6-year follow-ups.

**Methods:**

Longitudinal data of three subsequent measurements from the IDEFICS/I.Family cohort study were used to analyze the bi-directional association between moderate-to-vigorous PA (MVPA) and weight status by means of multilevel regression models. Analyses comprised 3393 (2-year follow-up) and 1899 (6-year follow-up) children aged 2–15.9 years from eight European countries with valid accelerometer data and body mass index (BMI) measurements. For categorized analyses, children’s weight status was categorized as normal weight or overweight (cutoff: 90th percentile of BMI) and children’s PA as (in-) sufficiently active (cutoffs: 30, 45 and 60 min of MVPA per day).

**Results:**

Children engaging in at least 60 min MVPA daily at baseline and follow-ups had a lower odds of becoming overweight (odds ratio [OR] at 2-year follow-up: 0.546, 95% CI: 0.378, 0.789 and 6-year follow-up: 0.393, 95% CI: 0.242, 0.638), compared to less active children. Similar associations were found for 45 min MVPA daily. On the other side, children who became overweight had the lowest odds to achieve 45 or 60 min MVPA daily (ORs: 0.459 to 0.634), compared to normal weight children.

**Conclusions:**

Bi-directional associations between MVPA and weight status were observed. In summary, at least 60 min MVPA are still recommended for the prevention of childhood overweight. To prevent excess weight gain, 45 min MVPA per day also showed preventive effects.

## Background

About 20% of children and adolescents in developed and developing countries are overweight or obese [1–3]. Although some studies have found no further increase in prevalence in recent years, the proportion of children and adolescents having overweight and obesity has reached a plateau at a high level [1, 4]. Childhood obesity and overweight are a major public health problem [2, 4] since cardiometabolic health is adversely affected by excessive fat mass [5, 6]. Furthermore, higher fat mass in childhood increases the risk of having obesity in adolescence and adulthood [7, 8]. The assumption that sufficient MVPA prevents excessive fat mass has become widely accepted since numerous cross-sectional studies have shown an inverse association between the level of high physical activity (PA) intensities such as moderate-to-vigorous PA (MVPA) and the prevalence of overweight and obesity in children [8–12]. Hence, national and international health care institutions recommend MVPA of at least 60 min per day for children and adolescents and 150 min of MVPA per week for adults [13, 14] to prevent obesity and other chronic diseases and to maintain metabolic health.

However, causal inferences about the direction of the association between PA and weight status can only be drawn from longitudinal studies using objective measurements of PA and age- and sex-adjusted body weight [15–17]. Up to now, only few longitudinal studies have been conducted with appropriate study designs [10, 15, 18]. Most of them with only small sample sizes (*N* < 300), either confirming the well-known cross-sectional association between PA and weight status [10] or finding an inverse association between body fat at baseline and MVPA at follow-up [15]. Furthermore, a recent review highlighted that “dose-response evidence” of PA for the prevention of childhood overweight and obesity is still lacking [19] and the recommended MVPA cutoff (60 min MVPA per day) supposed to prevent metabolic diseases such as obesity need to be scrutinized. This issue was also raised recently by Warburton and Bredin (2018) highlighting that dose-response-relationships in health studies are not always translated correctly. The authors pointed out that while some of the recommended health-improving doses of PA such as the MVPA level of 60 min per day may prevent chronic diseases, smaller amounts of PA may also improve several health parameters to an appreciable extent [20]. Our study offers the unique opportunity to investigate the bi-directional association between objectively measured MVPA and weight status in a large international sample (the IDEFICS/I.Family cohort), and moreover, to exploratively investigate whether MVPA less than the generally recommended 60 min per day (by arbitrarily taking 30 and 45 min per day as cutoff for sufficient MVPA) may already have a positive effect on weight status of children.

## Methods

### Study population

More than 16,000 participants aged 2–9.9 years were initially recruited at baseline (T0: 2007–2008) across eight European countries (Belgium, Cyprus, Estonia, Germany, Hungary, Italy, Spain, and Sweden) in the IDEFICS study. This study is currently the largest prospective study in European children investigating health-related behavior and metabolic outcomes in children and their families. Comprehensive examinations included physical examinations, blood samples, assessment of dietary and PA patterns at baseline, the first follow-up (T1: 2009–2010) and the second follow-up (T3: 2013–2014). It needs to be acknowledged that a further in-between follow-up (T2) was conducted as mail survey only and thus, was not considered in the present study. Parents gave written informed consent. Additionally, oral consent was obtained from children just before the examination. Ethical approval was obtained from the responsible authority of each participating study center. Further details of the study design and examination protocol have been reported elsewhere [21, 22].

### PA measurement

Intensities of PA were assessed by uniaxial accelerometry using Actigraph models (GT1M, GT3X and ActiTrainer; Actigraph, Pensacola, Florida, USA). The sample interval was set to an epoch of 15 s. During the first wave in Italy, an epoch length of 60 s was selected in most cases. We accounted for this by using the correction factor recommended by Colley and colleagues [23]. The accelerometers were attached to the right hip with a measuring tape. Each child was verbally instructed to wear the device for at least three consecutive days during waking hours, except when bathing or swimming. Additionally, all parents received written instructions on accelerometer usage and on how to record non-wear time of the device in diaries provided by the study personnel. About 13% of all children took off the device at least once during sports or water-based activities and recorded this in the non-wear diaries. If the recorded activities qualified as MVPA according to the Ainsworth Compendium for Youth [24], we imputed the recorded duration as MVPA as recently recommended [25], corrected by a factor of 0.5 for *all* different types of sports activities (physical education, training and competitions). Evenson cutoff-points [26] typically applied for school-aged children were used to categorize MVPA for all children (school-aged, kindergarten/pre-school children). Non-wear time was defined as at least 90 min of consecutive zeros and was removed from the data, as recommended [27]. To be included in the analysis, a minimum of six hours wear-time per day for at least 3 days (at least two weekdays and one weekend day) was required. Children with at least an average of 60 min MVPA per valid day were categorized as sufficiently active according to the WHO recommendations [13]. We consciously decided to use the absolute minutes of daily MVPA rather than proportions (%/day), since PA guidelines are also expressed in minutes per day.

### Anthropometric measurements

All physical examinations were conducted by trained study personnel according to the IDEFICS/I.Family study protocol in all three waves (T0/T1/T3). Waist circumference as well as body height and weight were measured at the local study centers. Details of the anthropometric measurements have been described elsewhere [22, 28]. Anthropometric values were included as percentiles for waist circumference and BMI, respectively.

#### Waist circumference

Waist circumference was measured in a standing position using a measuring tape (Seca 200), precision 0.1 cm, range: 0–150 cm.

#### Body mass index (BMI)

To calculate the BMI, body weight (kg) was divided by the square of body height (m). Children wore underwear and a T-shirt for weighing. Weight was measured to the nearest 0.1 kg using an electronic scale (Tanita BC 240 SMA, Tanita Europe, Sindelfingen, Germany). Height was measured barefoot with a telescopic height measuring instrument (Seca 225 stadiometer, Seca, Birmingham, UK) to the nearest 0.1 cm.

### Statistical analyses

Two different modeling approaches were chosen to examine the bi-directional relationship between PA and weight status. In the first approach, the outcome was modeled as a continuous variable and in the second approach as a dichotomous variable.

• Weight status as outcome:

Weight status of children was modeled continuously by means of BMI percentile based on the International (IOTF) BMI percentile curves updated by Cole & Lobstein [29]. For the second approach, this continuous variable was dichotomized and children with a BMI above the 90th percentile were considered having overweight or obesity.

• PA as outcome:

PA was modeled continuously as objectively measured average daily minutes of MVPA. For the second approach, this variable was dichotomized at the generally recommended MVPA cutoff, i.e., 60 min per day.

• Weight status / PA as categorized exposures:

When analyzing the potential influence of weight status on PA, weight status was modeled as a dichotomous exposure (as previously described) and when analyzing the possible influence of PA on weight status, PA was also modeled (as previously defined) as a dichotomous exposure. Finally, we combined exposure levels at baseline and follow-up to analyze the association of changes in weight status (between baseline and follow-up) on PA level at follow-up and vice versa, i.e., to analyze the association of changes in PA levels (between baseline and follow-up) on weight status at follow-up.

However, to explore whether even smaller amounts than 60 min daily are efficient to prevent overweight and obesity, we arbitrarily reduced this cutoff to also 45 and 30 min per day to categorize children as ‘sufficiently active’ in minutes of MVPA.

Based on these variables and cutoffs, associations between MVPA and weight status were analyzed by means of multilevel linear and logistic regression models (with country as random effect). Each of these approaches allowed to investigate both directions, i.e. the effect of the trajectory of PA (from baseline to follow-up) on weight status at follow-up (adjusted for baseline values) and vice versa. All regression estimates (beta coefficients, odds ratios and 95% confidence intervals (95% CI)) were determined using the SAS procedures MIXED and GLIMMIX (V9.3; SAS Institute Inc., Cary, North Carolina, USA), additionally adjusted for the duration between baseline wave (T0) and follow-up (T1/T3), age, sex, and socio-economic status (SES), the latter based on the “International Standard Classification of Education (ISCED)” contrasting low (ISCED levels 0–2) and medium (ISCED levels 3–5) parental education against high (ISCED levels 6–8) parental education as a proxy for SES [30]. In sensitivity analyses all regression analyses were repeated using the study-specific percentiles of waist circumference (instead of BMI percentiles) according to Nagy and colleagues [31]. Moreover, we evaluated whether observed associations differed for boys and girls or by region (North: Germany, Belgium, Sweden, and Estonia; South: Spain, Italy, Cyprus, and Hungary) within Europe. For the sake of simplicity, we will use the term ‘overweight’ instead of overweight/obesity in the following.

## Results

**Fig. 1:**
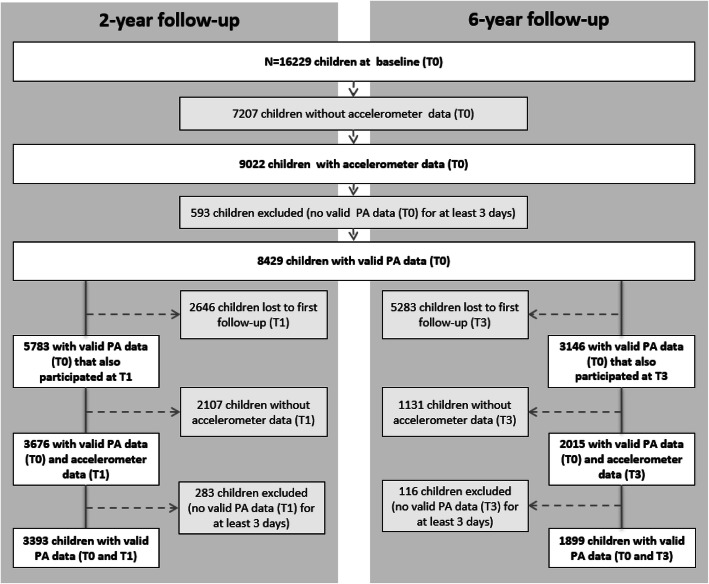
Study sample and exclusion criteria. Abbreviation: T0, baseline wave; T1, 2-year follow-up; T3, 6-year follow-up; PA, physical activity

Of 16,229 initially participating children at baseline, PA of more than nine thousand children was measured by accelerometry and valid PA was available in 8429 children (Fig. 1). Amongst those, 3393 children were included for the final analysis of the 2-year follow-up (providing valid PA data at both, T0 and T1). Regarding the analysis of the association between baseline and the 6-year follow-up a total of 1899 children could be included (providing valid PA data at both, T0 and T3).

About 50% of all children included in the 2-year and 6-year follow-up were male (Table 1). In both follow-ups, Sweden, Belgium and Cyprus provided the lowest and Estonia and Spain the largest number of valid observations. Only a small proportion of the children included had a low parental education (3.4–6.0%). The average age of all included children ranged between 5.9 and 6.3 years at baseline.

**Table 1:** Sociodemographic and anthropometric data, physical activity variables for 2-year and 6-year follow-up

	2-year follow-up (***N*** = 3393)				6-year follow-up (***N*** = 1899)			
	Boys (***N*** = 1692)		Girls (***N*** = 1701)		Boys (***N*** = 946)		Girls (***N*** = 953)	
**Country**	**N**	**%**	**N**	**%**	**N**	**%**	**N**	**%**
Italy	178	10.5	176	10.3	139	14.7	112	11.8
Estonia	346	20.4	372	21.9	219	23.2	216	22.7
Cyprus	73	4.3	73	4.3	25	2.6	31	3.3
Belgium	127	7.5	126	7.4	32	3.4	35	3.7
Sweden	77	4.6	90	5.3	63	6.7	57	6.0
Germany	256	15.1	250	14.7	120	12.7	149	15.6
Hungary	161	9.5	154	9.1	177	18.7	176	18.5
Spain	474	28.0	460	27.0	171	18.1	177	18.6
**Parental education (ISCED levels)**								
Missing	5	0.3	1	0.1	3	0.3	3	0.3
Low (levels 0–2)	101	6.0	102	6.0	32	3.4	32	3.4
Medium (levels 3–5)	770	45.5	742	43.6	402	42.5	416	43.7
High (levels 6–8)	816	48.2	856	50.3	509	53.8	502	52.7
**Age (years)**	**Mean**	**SD**	**Mean**	**SD**	**Mean**	**SD**	**Mean**	**SD**
Age at baseline	6.1	1.8	6.3	1.7	5.9	1.8	6.1	1.8
Age at follow-up	8.1	1.8	8.2	1.8	11.7	1.8	11.9	1.8
**BMI percentile**^a^								
BMI (PCTL.) at baseline	57.3	29.6	59.0	29.1	56.3	29.5	58.1	28.9
BMI (PCTL.) at follow-up	60.2	30.0	60.3	29.9	64.5	28.5	62.2	29.0
**Accelerometer wear time (min./day)**								
Wear time at baseline	704.5	120.4	699.6	126.1	708.8	137.4	707.3	135.4
Wear time at follow-up	695.8	110.1	698.5	109.5	754.9	114.5	757.3	101.3
**MVPA (min./day)**								
MVPA at baseline	55.5	22.5	47.6	19.9	53.4	22.9	46.2	19.3
MVPA at follow-up	57.6	25.0	48.2	21.7	58.5	24.6	47.9	20.4

*Abbreviations*: *ISCED* International Standard Classification of Education, *BMI* body mass index, *MVPA* moderate-to-vigorous physical activity

^a^ Based on the (IOTF) BMI percentile curves updated by Cole & Lobstein [29]

Across all waves, the average wear time of accelerometers was at least eleven hours and the time spent in MVPA per day ranged from 53 to 58 min in boys and 46 to 48 min in girls.

### Association between MVPA levels and subsequent weight status

The longitudinal association between MVPA levels on subsequent weight status was analyzed according to different trajectories of MVPA levels (i.e. the change of MVPA levels from baseline to follow-up) as exposure for the change in BMI percentiles at 2-year and 6-year follow-up (reference category: insufficiently active at baseline and follow-up) by varying MVPA cutoffs (< 30, 45 and 60 min per day, Table 2). At 2-year follow-up, the proportion of children who were categorized as sufficiently active (≥30, 45 and 60 min MVPA) at baseline and follow-up, decreased continuously ranging from 75% for the 30-min MVPA cutoff to 15% for the 60-min MVPA cutoff. Similarly, 74 and 12% of the children were found to be sufficiently active for the 30- and 60-min MVPA cutoffs, respectively, at 6-year follow-up.

**Table 2:** Association between combinations of different MVPA levels at baseline and follow-up with BMI percentile at 2-year (*N* = 3393) and 6-year follow-up (*N* = 1899)

Exposure: MVPA categories^**c**^	BMI percentile^a^ at 2-year follow-up			BMI percentile^a^ at 6-year follow-up		
	N (%)	β^**b**^ (95% CI)	***p***-value	N (%)	β^**b**^ (95% CI)	***p***-value
**Cutoff: 30 min./day** (baseline / follow-up)						
Ref. - / -	176 (5.2%)	**0**	**/**	65 (3.4%)	0	**/**
- / +	307 (9.0%)	0.229 (−2.876;3.335)	0.879	241 (12.7%)	1.780 (−4.179;7.738)	0.541
+ / -	359 (10.6%)	1.288 (−1.679;4.256)	0.377	195 (10.3%)	3.541 (−2.533;9.615)	0.239
+ / +	2551 (75.2%)	0.337 (−2.252;2.926)	0.789	1398 (73.6%)	−0.265 (−5.728;5.197)	0.921
**Cutoff: 45 min./day** (baseline / follow-up)						
Ref. - / -	828 (24.4%)	0	/	434 (22.9%)	0	**/**
- / +	652 (19.2%)	0.120 (−1.614;1.853)	0.887	466 (24.5%)	**−3.390 (−6.298;-0.482)**	0.025
+ / -	558 (16.4%)	0.114 (−1.683;1.912)	0.896	319 (16.8%)	1.595 (−1.572;4.762)	0.307
+ / +	1355 (40.0%)	−1.195 (−2.712;0.323)	0.117	680 (35.8%)	**−4.511 (−7.273;-1.749)**	0.003
**Cutoff: 60 min./day** (baseline / follow-up)						
Ref. - / -	1772 (52.2%)	**0**	**/**	987 (52.0%)	0	**/**
- / +	622 (18.3%)	−0.374 (−1.914;1.165)	0.618	402 (21.2%)	**−3.925 (−6.512;-1.338)**	0.005
+ / -	491 (14.5%)	−0.538 (−2.229;1.153)	0.516	289 (15.2%)	−0.532 (−3.431;2.367)	0.707
+ / +	508 (15.0%)	−1.266 (−2.957;0.425)	0.135	221 (11.6%)	**−4.184 (−7.461;-0.908)**	0.015

*Abbreviations*: *BMI* body mass index, *MVPA* moderate-to-vigorous physical activity, *CI* confidence interval

^a^Based on the (IOTF) BMI percentile curves updated by Cole & Lobstein [29]

^b^Estimates were adjusted for age, sex, parental education, baseline BMI percentile, duration between baseline and follow-up survey, and country

^c^**+**: MVPA ≥ cutoff (minutes per day), **−:** MVPA < cutoff (minutes per day)

During the 2-year follow-up, the BMI percentile at follow-up was not significantly affected by different MVPA trajectories. Only children who engaged at least 45 or 60 min in MVPA at baseline and 2-year follow-up, tend to have lower BMI percentiles at 2-year follow-up (β: -1.195, 95% CI: − 2.712, 0.323 and β: -1.266, 95% CI: − 2.957, 0.425) than children who did never achieve the 45 or 60 min MVPA per day. During 6-year follow-up, children who did engage less than 45 or 60 min at baseline but became sufficiently active at follow-up (≥45 or 60 min per day), had negative β-coefficients of − 3.390 (95% CI: − 6.298,-0.482) and − 3.925 (95% CI: − 6.512,-1.338) for the 45-min and 60-min MVPA cutoff, respectively, compared to children who were insufficiently active at baseline and 6-year follow-up, respectively. The strongest association between MVPA on BMI percentiles at follow-up was found in children who engaged at least 45 min per day at baseline and 6-year follow-up (β: -4.511, 95% CI: − 7.273,-1.749).

**Fig. 2:**
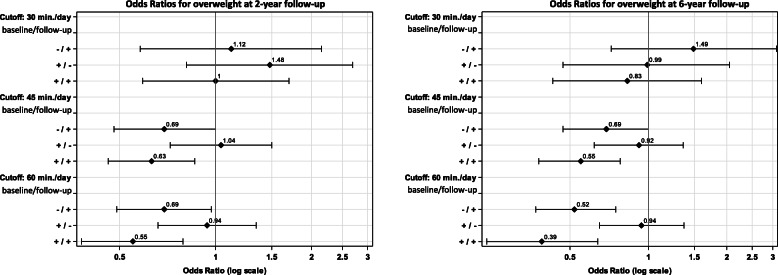
Association between combinations of different MVPA levels^a^ at baseline and follow-up and being overweight^b^ at 2-year follow-up (*N =* 3393) and at 6-year follow-up (*N =* 1899) in terms of odds ratios and 95%-confidence intervals. Abbreviations: MVPA, moderate-to-vigorous physical activity. ^a^ **+**: MVPA ≥ cutoff (minutes per day)), **−:** MVPA < cutoff (minutes per day). ^b^ > 90th percentile based on the (IOTF) BMI percentile curves updated by Cole & Lobstein [29]. ^c^ Estimates were adjusted for age, sex, parental education, baseline BMI percentile, duration between baseline wave and follow-up, and country, whereas the reference group comprised all children with MVPA below the corresponding cutoff (minutes per day) at both waves (− / -)

Analogously, the odds ratios for becoming overweight at 2-year and 6-year follow-up were calculated for the different MVPA cutoffs (30, 45 and 60 min per day). At both follow-ups, the lowest odds for becoming overweight was observed in children who were sufficiently active at baseline *and* follow-up - regardless of the MVPA cutoff (Fig. 2).

Compared to children who were insufficiently active in both waves (ref.), the odds for becoming overweight at 2-year follow-up was 31% lower in children who were insufficiently active at baseline but became sufficiently active at follow-up for the 45-min MVPA cutoff as well as for the 60-min MVPA cutoff (Fig. 2). Even smaller odds ratios for becoming overweight at 2-year follow-up were found in children who were sufficiently active in both waves (45-min cutoff: OR = 0.628; 60-min cutoff: OR = 0.546).

At the 6-year follow-up, the lowest odds for becoming overweight has been found in children who were sufficiently active at both waves with an OR of 0.545 (95% CI: 0.382, 0.779) for the 45-min MVPA cutoff and an OR of 0.393 (95% CI: 0.242, 0.638) the 60-min cutoff (as compared to children who did not achieve the 45 or 60 min MVPA per day, neither at baseline nor at follow-up). The odds for becoming overweight at 6-year follow-up in children who were insufficiently active at baseline but became sufficiently active at follow-up, was approximately 31% lower for the 45-min MVPA cutoff (OR: 0.686, 95% CI: 0.471, 0.999) and 48% lower for the 60-min MVPA cutoff (OR: 0.524, 95% CI: 0.365, 0.752), compared to children who were insufficiently active in both waves. Tables 3 and 4 in the Annex show all odds ratios and 95% confidence intervals graphically presented in Fig. 2.

**Table 3:** Association between combinations of different MVPA levels at baseline and follow-up and being overweight at 2-year (*N =* 3393) and 6-year follow up (*N =* 1899) in terms of prevalence of overweight, odds ratios and 95%-confidence intervals

Exposure: MVPA categories^**a**^	Prevalence and odds ratios for becoming overweight at 2-year follow-up				Prevalence and odds ratios for becoming overweight at 6-year follow-up			
	N	%^b^	OR^**c**^	95% CI	N	% ^b^	OR^**c**^	95% CI
**Cutoff: 30 min./day** (baseline / follow-up)								
Ref. - / -	176	32.4%	**0**	**/**	65	33.8%	0	/
- / +	307	19.5%	1.119	0.582;2.152	241	31.5%	1.492	0.718;3.102
+ / -	359	39.0%	1.479	0.814;2.687	195	29.2%	0.986	0.474;2.052
+ / +	2551	21.1%	1.003	0.592;1.698	1398	22.0%	0.829	0.430;1.600
**Cutoff: 45 min./day** (baseline / follow-up)								
Ref. - / -	828	31.6%	0	/	434	32.7%	0	/
- / +	652	18.6%	0.692	0.477; 1.003	466	23.2%	**0.686**	0.471;0.999
+ / -	558	28.0%	1.043	0.724; 1.504	319	27.0%	0.915	0.617;1.359
+ / +	1355	18.8%	**0.628**	0.460; 0.859	680	18.7%	**0.545**	0.382;0.779
**Cutoff: 60 min./day** (baseline / follow-up)								
Ref. - / -	1772	27.4%	0	/	987	29.9%	0	/
- / +	622	19.0%	**0.694**	0.496;0.970	402	16.9%	**0.524**	0.365;0.752
+ / -	491	22.4%	0.936	0.655;1.337	289	24.2%	0.941	0.647;1.367
+ / +	508	15.9%	**0.546**	0.378;0.789	221	13.6%	**0.393**	0.242;0.638

*Abbreviations*: *MVPA* moderate-to-vigorous physical activity, *OR* odds ratio, *CI* confidence interval

^**a**^**+**: MVPA ≥ cutoff (minutes per day), **−:** MVPA < cutoff (minutes per day)

^b^> 90th percentile based on the (IOTF) BMI percentile curves updated by Cole & Lobstein [29]

^**c**^Estimates were adjusted for age, sex, parental education, baseline BMI percentile, duration between baseline and follow-up survey, and country

**Table 4:** Association between combination of different weight status according to BMI percentiles at baseline and follow-up and sufficient engagement in MVPA according to different cutoffs at 2-year (*N* = 3393) and 6-year follow-up (*N* = 1899) in terms of prevalences of children reaching at least the cutoffs, odds ratios, and 95%-confidence intervals

	**Sufficient MVPA (min./day) at 2-year follow-up**									
**Exposure:** **Weight categories**		**Cutoff: ≥30 min**			**Cutoff: ≥45 min**			**Cutoff: ≥60 min**		
Baseline ➔ follow-up	**N**	**%**^**a**^	**OR**^**b**^	**95% CI**	**%**^**a**^	**OR**^**b**^	**95% CI**	**%**^**a**^	**OR**^**b**^	**95% CI**
NW ➔ NW (Ref.)	2512	87.0%	1	/	62.6%	1	/	35.7%	1	/
NW ➔ OW	249	81.9%	0.826	0.566;1.206	46.2%	**0.532**	0.398;0.711	25.3%	**0.634**	0.460;0.875
OW ➔ NW	87	86.2%	1.024	0.521;2.010	59.8%	0.967	0.598;1.564	36.8%	1.197	0.742;1.930
OW ➔ OW	545	71.9%	**0.728**	0.560;0.946	47.5%	0.864	0.695;1.074	25.0%	0.857	0.676;1.087
	**Sufficient MVPA (min./day) at 6-year follow-up**									
**Exposure:** **Weight categories**		**Cutoff: ≥30 min**			**Cutoff: ≥45 min**			**Cutoff: ≥60 min**		
Baseline ➔ follow-up	**N**	**%**^**a**^	**OR**^**b**^	**95% CI**	**%**^**a**^	**OR**^**b**^	**95% CI**	**%**^**a**^	**OR**^**b**^	**95% CI**
NW ➔ NW (Ref.)	1349	87.4%	1	/	63.4%	1	/	36.6%	1	/
NW ➔ OW	219	84.9%	0.793	0.510;1.234	53.0%	**0.588**	0.429;0.806	22.8%	**0.459**	0.322;0.655
W ➔ NW	87	87.4%	1.618	0.789;3.318	63.2%	1.373	0.834;2.262	35.6%	1.226	0.746;2.015
OW ➔ OW	244	81.1%	1.252	0.826;1.899	48.8%	0.870	0.636;1.190	19.7%	**0.624**	0.434;0.896

*Abbreviations*: *BMI* body mass index, *MVPA* moderate-to-vigorous physical activity, *OR* odds ratio, *CI* confidence interval, *OW* overweight children (>90th percentile of BMI), *NW* normal weight children (≤90th percentile of BMI) based on the (IOTF) BMI percentile curves updated by Cole & Lobstein [29]

^a^Prevalence of children reaching at least the corresponding cutoff

^b^Estimates were adjusted for age, sex, education, baseline MVPA, duration (years) between baseline and follow-up survey, and country

### Association between weight status and subsequent MVPA levels

The longitudinal association between weight status and subsequent MVPA levels was analyzed using different combinations of weight status at baseline and 2−/6-year follow-up, respectively as exposure for the change in MVPA at 2-year and 6-year follow-up compared to the reference category (normal weight at both baseline and follow-up), see Table 5.

**Table 5:** Association between the combinations of different weight status according BMI percentiles at baseline and follow-up with average duration of MVPA per day at 2-year (*N* = 3393) and 6-year follow-up (*N* = 1899)

Exposure: Weight categories (baseline ➔ follow-up)	MVPA (min./day) at 2-year follow-up			MVPA (min./day) at 6-year follow-up		
	N (%)	β^a^ (95% CI)	***p***-value	N	β^a^ (95% CI)	***p***-value
NW ➔ NW (Ref.)	2512 (74.0%)	0	/	1349 (71.0%)	0	/
NW ➔ OW	249 (7.3%)	**−4.612 (− 7.548;-1.676)**	0.004	219 (11.5%)	**− 7.512 (− 10.700;-4.323)**	<.001
OW ➔ NW	87 (2.6%)	0.917 (−3.887;5.721)	0.696	87 (4.6%)	1.790 (−3.055;6.635)	0.450
OW ➔ OW	545 (16.1%)	**−2.103 (−4.297;0.091)**	0.059	244 (12.8%)	**−3.657 (− 6.838;-0.475)**	0.026

*Abbreviations*: *BMI* body mass index, *MVPA* moderate-to-vigorous physical activity, *CI* confidence interval, *OW* overweight children (>90th percentile of BMI), *NW* normal weight children (≤90th percentile of BMI) based on the (IOTF) BMI percentile curves updated by Cole & Lobstein [29]

^a^Estimates were adjusted for age, sex, parental education, baseline MVPA, duration between baseline and follow-up survey, and country

Most children remained categorized as either normal weight (2-year follow-up: 74% (2512/3393); 6-year follow-up: 71% (1349/1899)) or overweight (2-year follow-up: 16% (545/3393); 6-year follow-up: 13% (244/1899) during follow-ups. In contrast, only 2.6% (T1) and 4.6% (T3) changed from overweight to normal weight and 7.3% (T1) and 11.5% (T3) became overweight in the time between baseline and follow-ups. In particular, children who were normal weight at baseline but overweight at the 6-year follow-up, decreased their MVPA per day by on average 7.5 min (95% CI: − 10.700,-4.323) on average compared to the reference category (normal weight at baseline and follow-up).

Analogously to the approach described above, odds ratios for being sufficiently active according to the three different MVPA cutoffs (≥30/45/60 min per day) at 2-year and 6-year follow-up were calculated for different weight trajectories at baseline and follow-up.

**Fig. 3:**
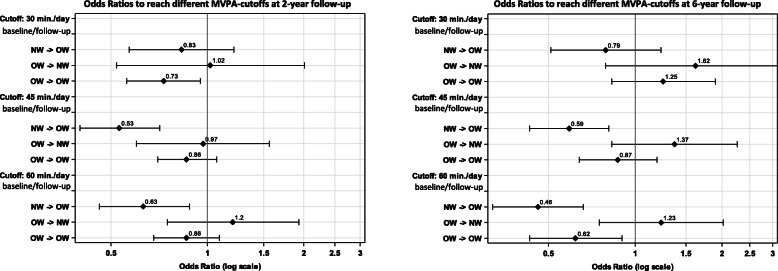
Association between the combination of different weight status according to BMI percentiles at baseline and follow-up and sufficient engagement according to different cutoffs in MVPA at 2-year follow-up (*N* = 3393) and at 6-year follow-up (*N* = 1899) in terms of odds ratios and 95%-confidence intervals^a^. Abbreviations: BMI, body mass index; MVPA, moderate-to-vigorous physical activity; CI, confidence interval; OW, overweight children (>90th percentile of BMI); NW, normal weight children (≤90th percentile of BMI) based on the (IOTF) BMI percentile curves updated by Cole & Lobstein [29]. ^a^ Estimates were adjusted for age, sex, parental education, baseline MVPA, duration between baseline wave and follow-up, and country, whereas the reference group comprised all children who were normal weight at both waves (NW- > NW)

Regarding to the 30-min cutoff, the most notable result was that children who were overweight at both waves had a 27% decreased odds to even achieve 30 min MVPA per day at 2-year follow-up (Fig. 3). Regarding the aim to reach at least 45 or 60 min MVPA per day, the lowest odds was observed in both follow-ups for children who changed their weight status from normal weight (baseline) to overweight (follow-up) with ORs of 0.63 (2-year follow up) and 0.46 (6-year follow up) as compared to normal weight children, that did not change their weight status (Fig. 3). Moreover it was seen, that children who were overweight at baseline and 6-year follow-up, respectively, had a 38% decreased odds to be sufficiently active according the WHO recommendation of 60 min MVPA per day (OR: 0.624; 95%-CI: 0.434, 0.896), compared to the reference category of children of normal weight at both baseline and 6-year follow-up (Fig. 3).

### Sensitivity analyses

As different markers for weight status have been used in studies investigating associations between PA and overweight, we also ran our analyses with percentiles of waist circumference. We found only small discrepancies in terms of smaller/wider confidence intervals, but neither did the direction of association change nor did appear or disappear. Moreover, we did not reveal notable differences either regarding different regions (North vs. South), or for boys and girls.

## Discussion

The present study aimed to investigate the bi-directional association between objectively measured MVPA and weight status in a large sample, and to analyze whether less than 60 min MVPA per day may also prevent childhood overweight. To the best of our knowledge, until now, a solid data base (i.e. reliable measurements in a large longitudinal sample of children) for analyzing the association of changes in PA levels and weight status (and vice versa) was missing. Regarding the first aim, our results showed that children being sufficiently active at baseline and follow-up reduced the odds of becoming overweight at follow-up and that being overweight was associated with a lower odds of achieving the generally recommended MVPA levels in the following years of childhood. In particular, our data showed that the MVPA cutoff recommended by the WHO (at least 60 min per day) is evident regarding the prevention of childhood overweight. However, concerning the second aim, we found a comparable protective effect even for a threshold of 45 min daily MVPA. Therefore, future studies promoting MVPA should target to motivate children to engage in more MVPA as before. To optimally prevent excess weight gain, 60 min MVPA per day are still recommended. Whilst the latter might be hard to achieve, particularly for overweight children, those children should be motivated to engage at least 45 min in MVPA – without losing the preventive effect.

We further found that only a small proportion of children who were overweight at baseline became normal weight during the following 2 or 6 years (2.6 and 4.6%, respectively). In contrast, more normal weight children at baseline were overweight at follow-up (2-year follow-up: 7.3%, 6-year follow-up: 11.5%, Table 5). Thus, once overweight was established, it was harder to reverse this development since high MVPA levels shown to be preventive for overweight were hard to achieve for overweight children. In summary, the observed bi-directional association between MVPA and BMI highlight a vicious circle regarding excess weight gain. On a positive note, the number of children who were insufficiently active at baseline but became sufficiently active at both follow-ups was higher than the number of children who were active enough at baseline but insufficiently active at follow-up, regardless whether the MVPA cutoff was 45 or 60 min per day.

We carried out several sensitivity analyses to evaluate whether the results may be influenced e.g. by the definition of weight status, or to check for robustness of results by stratifying the data by sex or region (since recent studies reported that children from Northern Europe were more likely to achieve the recommended MVPA level [60 min MVPA per day] compared to children living in Southern Europe [32]). However, none of these approaches yielded significant results that differed from the central findings of this study.

### Comparing the present findings with other studies

Comparing our results was challenging since, to our knowledge, no study analyzed bi-directional associations between objectively measured MVPA and BMI in such a large international sample so far. Despite the large data base, cross-country comparisons were not possible due to loss of observations in each country. In general, our results were in line with the consistent findings of recent studies which found that PA levels were lower in children with overweight compared to normal weight children and that boys accumulate more MVPA than girls (Table 1) [17, 33, 34].

Although a growing number of longitudinal studies on obesity used objective PA measures in recent years [15, 16, 18, 33, 35–38], results were inconsistent regarding the association between PA and weight status. One study investigated the bi-directional association between PA and weight status and reported no association in school-aged children [35]. Another longitudinal study did not observe any association between baseline PA and the change in body fat percentage at follow-up but a statistically significant relationship between baseline body fat and PA at follow-up (*r =* − 0.16 to − 0.22) [15]. In a further study, neither MVPA nor sedentary behavior predicted a higher waist circumference at follow-up, but higher baseline waist circumference was associated with increased sedentary behavior at follow-up [39]. North-East England, a 2-year follow-up study showed that the decline in MVPA was associated with greater increases of fat mass index and BMI z-scores in 7-year-old boys, but not in girls of the same age [36]. The authors hypothesized that PA may have a higher impact on overweight in boys than in girls. In the USA, a 6-year follow-up study reported that MVPA at the age of 9 years was negatively associated with BMI at the age of 15 years in boys and girls having overweight (90th BMI percentile). In the same study, increased sedentary behavior was associated with increases of BMI 6 years later, independent of the amount of MVPA. The authors concluded that decreasing sedentary behavior and increasing MVPA may have independent beneficial effects on the prevalence of overweight [37]. In contrast, another study from the USA reported that the association between sedentary behavior and BMI z-score in preschool children disappeared after adjustment for MVPA [38]. A 5-year follow-up study (*N* = 600) also conducted at three different time points and in five European countries (Germany, Italy, Poland, Belgium, and Spain) found a bi-directional association between PA and weight status in 430 children having at least two measurements of objectively measured PA and BMI [40]. This seems particularly interesting, since the study used another device and different sampling intervals to measure PA (Sensewear Armband with an epoch of 60 s), but, however, their results also suggest a bi-directional relationship between weight status and PA. The authors particularly highlighted the role of sedentary behavior regarding childhood obesity, since they found the positive association between sedentary behavior and BMI to increase with age. Contrary to our study, PA was included without considering current PA guidelines and hence, results are therefore not directly comparable with ours.

### Strengths and limitations

One important strength of the present study is the large sample size (2-year follow-up *N* = 3393; 6-year follow-up *N* = 1899) used to investigate the association between different trajectories of MVPA levels and different trajectories of weight status. Furthermore, we provide specific dose-response relationships of different MVPA cutoffs facilitating the recommendation of specific MVPA levels for the prevention of childhood overweight. Regarding PA, the used accelerometers are commonly used to quantify the amount and intensity of PA [41] and the applied cutoff points by Evenson and colleagues are recommended for young children [26]. Further, to account for times when children took off the device during sports activities we improved accelerometer data by imputing MVPA (if applicable) as recommended [25]. To accurately assess the sporadic PA pattern in children, accelerometer sampling intervals shorter than five seconds are recommended [42]. Thus, the applied sampling intervals (15 to 60 s) used in this study is a limitation we need to acknowledge. At least, to account for the well-known underestimation associated with the use of PA data based on longer epoch settings [43], we used the correction factor recommended by Colley and colleagues [23] for the epoch setting of 60 s selected in most cases in Italy during the first wave.

However, our study has a clear limitation - the attrition of the study sample is a common problem of longitudinal studies [33]. Similar to our observation, other studies reported a high attrition during the 6-year follow-up period, leading to a downsized study population of about one third [33, 37]. There are also limitations regarding the PA assessment in this study. Although a minimum accelerometer wear time of four to five days [9] and ten hours have recently been recommended [37, 44], three days and six hours have been reported to provide acceptable reliability of total PA, MVPA, and total sedentary time [45]. Another limitation is the use of different Actigraph accelerometers, since inconclusive findings have been found in validation studies. Whereas some study reported that different Actigraph devices provided comparable measured counts across all PA intensities [46, 47], one study highlighted differences between Actigraph models particular for lower intensities such as light PA and sedentary time [48]. However, light PA and sedentary time have not been addressed in the present study, since this would have gone beyond the scope of the present study. Finally, with regard to representativeness of the data base it must be stressed that our study did not intended to be strictly nationally representative. Nevertheless, the study provides population-based samples that were drawn from communities considered to be typical of their geographical region.

## Conclusion

An inverse, bi-directional association between objectively measured MVPA levels and BMI in children was observed for the generally recommended 60 min MVPA per day, but also for an even lower MVPA cutoff of 45 min. While sufficient MVPA (45 or 60 min per day) prevent overweight in later childhood, such high MVPA levels are hard to achieve by children having overweight. This highlights a vicious circle that should be considered in primary prevention of childhood overweight and obesity. In summary, at least 60 min MVPA are still recommended for the prevention of childhood overweight, but, if that is not possible, 45 min might also be sufficient. However, more high-quality studies, i.e., longitudinal studies with objectively and standardized measurements of weight status and PA, are needed to support our findings.

## Data Availability

The datasets generated and analyzed during the current study are not publicly available because this study is based on highly sensitive data collected in young children. But interested researchers can contact the IDEFICS and I. Family consortia (http://www.ideficsstudy.eu/Idefics/ and http://www.ifamilystudy.eu/) to discuss possibilities for data access.
